# Leucine-Rich α2 Glycoprotein as a Predictor of Small Bowel Disease Activity in Crohn’s Disease: A Retrospective Study

**DOI:** 10.31662/jmaj.2025-0355

**Published:** 2025-12-05

**Authors:** Tomoyuki Hayashi, Kazuya Kitamura, Masaaki Usami, Noriaki Orita, Hidetoshi Nakagawa, Masaki Miyazawa, Hajime Takatori, Masaki Nishitani, Akihiro Dejima, Tetsuro Shimakami, Kosuke Satomura, Makiko Kimura, Hirofumi Okafuji, Hiroto Saito, Daisuke Yamamoto, Noriyuki Inaki, Tadashi Toyama, Taro Yamashita

**Affiliations:** 1 Inflammatory Bowel Disease Center, Kanazawa University Hospital, Kanazawa, Japan; 2 Department of Gastroenterology, Kanazawa University, Kanazawa, Japan; 3 Department of Pediatrics, Kanazawa University, Kanazawa, Japan; 4 Department of Gastrointestinal Surgery, Kanazawa University, Kanazawa, Japan; 5 Innovative Clinical Research Center, Kanazawa University, Kanazawa, Japan; 6 Department of Nephrology, University of Fukui, Fukui, Japan

**Keywords:** Crohn’s disease, Leucine-rich α-2 glycoprotein, small bowel, balloon-assisted enteroscopy

## Abstract

**Introduction:**

Balloon-assisted enteroscopy is the gold standard for evaluating small bowel lesions in Crohn’s disease (CD); however, its invasiveness and cost limit routine use. Leucine-rich α-2 glycoprotein (LRG) has emerged as a potential noninvasive biomarker. This study aimed to assess the diagnostic utility of LRG compared with conventional biomarkers.

**Methods:**

This retrospective study included 216 patients with CD who underwent balloon-assisted enteroscopy between April 2021 and March 2024. Serum biomarkers, including LRG, C-reactive protein, leukocyte count, neutrophil count, hemoglobin, platelet count, erythrocyte sedimentation rate, and albumin, were analyzed. Endoscopic activity was defined as mucosal ulcers measuring ≥0.5 cm. Diagnostic performance was evaluated using receiver operating characteristic curve analysis, and predictors of endoscopic activity were identified using multivariate logistic regression. Prognostic value was assessed using hospitalization-free and surgery-free survival.

**Results:**

LRG demonstrated the highest diagnostic accuracy (area under the ROC curve (AUC), 0.906), outperforming C-reactive protein ( area under the receiver operating characteristic curve: 0.776). An LRG cutoff of 16.3 μg/mL yielded 72.1% sensitivity and 93.7% specificity. Elevated LRG was independently associated with endoscopic activity (odds ratio: 36.4, p < 0.001) and correlated with higher modified Simple Endoscopic Score for Crohn’s disease (mSES-CD). High LRG levels were also predictive of poorer hospitalization-free and surgery-free survival.

**Conclusions:**

LRG is a reliable and non-invasive biomarker for assessing small bowel disease activity in CD, showing superior diagnostic and prognostic performance compared with conventional biomarkers. It may be a valuable adjunct to endoscopic and imaging evaluations in clinical practice.

## Introduction

Crohn’s disease (CD) is a chronic inflammatory bowel disease (IBD) that can lead to strictures, fistulas, impaired quality of life, and the need for bowel surgery ^(1)^. Patients with small bowel or ileocecal involvement are at greater risk for surgery than those with colonic disease ^(2)^, making accurate assessment of small bowel lesions essential for optimal disease management.

A treatment-to-target approach is recommended for IBD, aiming at both clinical and endoscopic remission. Endoscopic remission, defined as the absence of mucosal inflammation on ileocolonoscopy, is the global standard for evaluating mucosal healing in CD. However, proximal small bowel lesions beyond the reach of ileocolonoscopy are common and clinically relevant. Balloon-assisted enteroscopy (BAE) provides direct evaluation and tissue sampling, but its invasiveness and limited availability restrict routine use. Capsule endoscopy enables assessment of the whole small bowel but carries a risk of retention, especially in patients with strictures. Magnetic resonance enterography (MRE) offers transmural evaluation but is costly and time-consuming, while bowel ultrasonography is noninvasive and bedside-available but operator-dependent ^(3)^. These limitations highlight the need for reliable, noninvasive biomarkers. In addition, comparative studies have demonstrated correlations between endoscopic indices and imaging modalities such as MRE, computed tomography enterography, and capsule endoscopy, which provide complementary information regarding small-bowel disease activity and complications ^(3), (4), (5)^.

In CD, small bowel involvement is common, with population-based cohorts reporting that more than half of patients develop small bowel lesions during the disease course ^(6)^. In Japan, the national cohort registry of newly diagnosed patients (iCREST-CD) demonstrated a high prevalence of small bowel involvement ^(7)^. The presence of active small bowel lesions is independently associated with poor prognosis in CD ^(8)^, underscoring the importance of early identification and management in therapeutic strategies. Currently used biomarkers such as C-reactive protein (CRP) and fecal calprotectin (FC) are widely applied as treatment targets ^(9), (10)^. Leucine-rich α-2 glycoprotein (LRG) has recently emerged as a novel serum biomarker ^(11)^. Initially identified in rheumatoid arthritis, LRG is induced by interleukin (IL)-22, tumor necrosis factor-α, and IL-1β, independent of IL-6, and has been shown to correlate with disease activity in ulcerative colitis ^(12), (13), (14), (15)^. In CD, LRG is upregulated and correlates more strongly with disease activity than CRP ^(11), (13), (16), (17), (18), (19), (20)^.

However, it remains uncertain whether LRG offers superior diagnostic utility for small bowel lesions compared with CRP and other markers, such as leukocytes, neutrophils, hemoglobin, platelets, erythrocyte sedimentation rate (ESR), and albumin. This study therefore aimed to evaluate LRG as a predictor of small bowel disease activity compared with conventional biomarkers in CD, and to investigate its association with adverse outcomes such as hospitalization and surgery.

## Materials and Methods

### Study population

This retrospective case-series study was conducted at Kanazawa University Hospital (Ishikawa, Japan) and included data from all consecutive patients with CD who underwent BAE between April 2021 (when LRG measurement became available at the hospital) and March 2024. All 216 patients fulfilled the diagnostic criteria outlined by the Japanese Ministry of Health, Labor, and Welfare ^(21)^. All patients underwent confirmation of their symptoms and biomarker assessment, including LRG, leukocytes, neutrophils, hemoglobin, platelets, CRP, ESR and albumin, on the same day they underwent BAE. A commercially available kit was used for LRG measurements (Nanopia LRG Kit, Sekisui Medical, Tokyo, Japan). The exclusion criteria were as follows: active ulcers predominantly in the colon; colostomy or ileostomy; intolerance or contraindications to BAE; and other diseases that could affect LRG and CRP levels, including extraintestinal complications, collagen disease, infectious diseases, and malignancy, as LRG is produced by neutrophils ^(22)^. If multiple BAEs were performed during the study period, only the first procedure was included, while the others were excluded. Written consent was obtained from all patients to publish their information.

### Assessment of endoscopic activity of CD

At our facility, double-balloon enteroscopy was performed using the two-operator method, with retrograde insertion as deep as possible in all cases. Insertion was terminated when large ulcers were detected to avoid complications such as perforation. Endoscopic evaluation was performed using the mSES-CD ^(20), (23)^. The modified SES-CD was calculated as the sum of the scores across the terminal ileum, proximal ileum, and jejunum, in accordance with previous reports. This approach was used to comprehensively evaluate small bowel disease activity rather than relying on the most severe lesion alone. The modified SES-CD excluded the “stenosis” score from the original SES-CD, which includes four endoscopic variables (ulcer size, proportion of ulcerated surface, proportion of affected surface, and stenosis) because the focus was on active disease. The modified SES-CD was determined through a consensus among two endoscopists and a supervising physician. “Endoscopic activity” was defined as the presence of a mucosal defect (ulcer) measuring ≥0.5 cm, as defined in the SES-CD. Anastomotic ulcers were included since the modified SES-CD does not distinguish ulcer etiology; their presence often indicates active inflammation and warrants similar therapeutic strategies. All procedures used retrograde double-balloon enteroscopy and were performed by four experienced endoscopists.

### Outcome measures

Patient background, including age, sex, and clinical characteristics such as the extent of disease involvement and biomarkers, was assessed. Receiver operating characteristic (ROC) analysis was performed for endoscopic activity, LRG, and other biomarkers to identify appropriate cutoff values and assess the diagnostic accuracy of each biomarker. The modified SES-CD was divided into groups 0-2, 3-4, 5-6, and ≥7. The LRG distribution in each group, and the percentage below the cutoff value, were assessed. Associations between endoscopic disease activity and patient background, biomarkers, and clinical activity, measured by the Crohn’s Disease Activity Index (CDAI), were examined to identify predictive parameters for endoscopic activity. To evaluate the utility of LRG in assessing endoscopic disease activity, a multivariate analysis was performed using endoscopic disease activity as the objective variable and biomarkers, including LRG, as explanatory variables. Explanatory variables included age, sex, biomarkers, and clinical activity. The LRG was analyzed using binary indicators (above or below the cutoff value) to validate the cutoff value. Outcome-free survival of hospitalization and surgery rates were compared between the high (above the cutoff value) and low (below the cutoff value) LRG groups.

Hospitalization was defined as any unplanned admission related to CD management, excluding elective procedures. Surgery was defined as intestinal resection or strictureplasty for CD-related complications. The median follow-up durations for hospitalization and surgery were 492 and 529 days, respectively.

### Statistical analyses

Continuous nonparametric variables are expressed as median with range or interquartile range (IQR), and parametric variables are expressed as mean with standard deviation. Between-group comparisons were performed using the Student’s *t*-test or the Mann-Whitney U test. Categorical variables are expressed as percentages, and between-group comparisons were performed using Fisher’s exact test. The association between endoscopic activity of CD and biomarkers was examined using ROC curve analysis and the area under the ROC curve (AUC). The point with the smallest distance from the upper-left corner was set as the cutoff value. Binomial logistic regression analysis was performed as a multivariate analysis to evaluate the utility of LRG in assessing endoscopic activity. Kaplan-Meier survival analysis was performed to compare hospitalization- and surgery-free survival rates, and the hazard ratio (HR) was calculated. Differences with p < 0.05 were considered to be statistically significant. All statistical analyses were performed using Prism version 10 (GraphPad Inc., San Diego, CA, USA).

## Results

### Patient characteristics

While 58.8% of patients were classified as L3 (ileocolonic CD), those included in the analysis did not exhibit active colonic ulcerations at the time of BAE. Seventeen L3 patients with active colonic ulcers were excluded from the study.

Biomarker analysis revealed a median LRG level of 15.9 μg/mL (range, 5.8-61.9). The median levels of the other biomarkers assessed were as follows: leukocytes (5,020/μL), neutrophils (3,110/μL), hemoglobin (13.4 g/dL), platelets (28.1 × 10^4/μL), CRP (0.14 mg/dL), ESR (13 mm/h), and albumin (4.0 g/dL) (Table 1).

**Table 1.** Baseline Characteristics.

Characteristic	Value	All patients (n = 216)
Median age, years (range)		40.5 (10-79)
Sex, females/males, n (%)		47 (21.8) : 169 (78.2)
CDAI, average		104.4 ± 71.7
Montreal classification, n (%)		
	Age at diagnosis, A1 : A2 : A3	34 (15.7) : 163 (75.5) : 19 (8.8)
	Location, L1 : L3	89 (41.2) : 127 (58.8)
	Behavior, B1 : B2 : B3	56 (25.9) : 96 (44.4) : 64 (29.6)
mSES-CD, average		3.96 ± 2.80
	0-2, n (%)	56 (25.9)
	3-4, n (%)	60 (27.8)
	5-6, n (%)	60 (27.8)
	7-9, n (%)	40 (18.5)
Biomarkers, median (range)		
	LRG, μg/mL	15.9 (5.8-61.9)
	Leukocyte count, /μL	5,020 (2080-14,560)
	Neutrophil count, /μL	3,110 (970-12,940)
	Hemoglobin, g/dL	13.4 (7.5-16.9)
	Platelet count, 10^4/μL	28.1 (3.9-68.4)
	CRP, mg/dL	0.14 (0-12.6)
	ESR, mm/h	13 (2-99)
	Albumin, g/dL	4.0 (1.7-5.0)

CDAI: Crohn’s disease activity index; CRP: C-reactive protein; ESR: erythrocyte sedimentation rate; LRG: leucine-rich α-2 glycoprotein.

A total of 114 patients (52.8%) had a history of intestinal resection. Among them, 42 had visible anastomotic ulcers, all of which occurred more than one year after surgery.

### Association between the endoscopic activity of CD and biomarkers, including LRG

**Figure 1.**
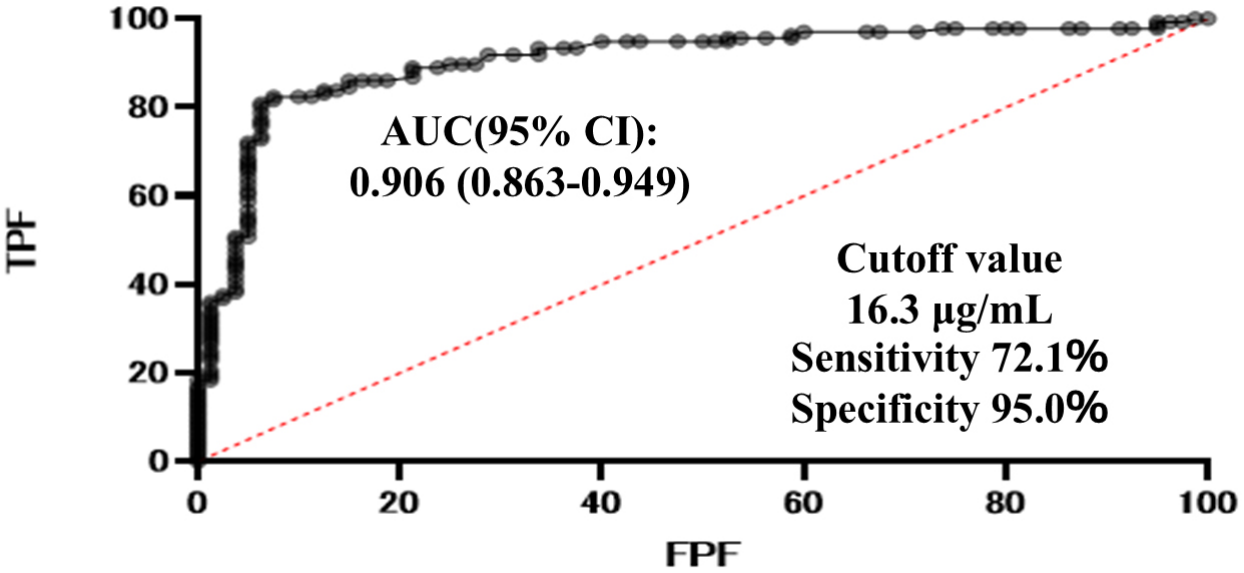
Diagnostic accuracy of LRG based on receiver operating characteristic curve analysis for endoscopic activity. AUC: area under the curve; CI: confidence interval; FPF: false positive fraction; LRG: leucine-rich α-2 glycoprotein; TPF: true positive fraction.

The ROC curve analysis of biomarker diagnostic accuracy revealed that the AUC for LRG was 0.906 (95% confidence interval [CI], 0.863-0.949) (Figure 1), which was higher than those for leukocytes (0.503 [95% CI, 0.422-0.584]), neutrophils (0.620 [95% CI, 0.542-0.698]), hemoglobin (0.633 [95% CI, 0.558-0.708]), platelets (0.594 [95% CI, 0.512-0.675]), CRP (0.776 [95% CI, 0.713-0.839]), ESR (0.679 [95% CI, 0.606-0.753]), albumin (0.785 [95% CI, 0.724-0.846]), and CDAI (0.734 [95% CI, 0.668-0.800]). Furthermore, the optimal LRG cutoff value (16.3 μg/mL) showed 72.1% sensitivity and 93.7% specificity (Table 2).

**Table 2.** Diagnostic Accuracy of Biomarkers Based on ROC Curve Analysis for Endoscopic Activity～ROC Analysis～.

Biomarker	AUC (95% CI)	Sensitivity (%, 95% CI)	Specificity (%, 95% CI)
LRG (μg/mL)	0.906 (0.863-0.949)	72.1 (64.0-78.9)	93.7(86.2-97.3)
Leukocyte(/μL)	0.503 (0.422-0.584)	59.6 (51.2-67.4)	46.3 (35.7-57.1)
Neutrophil (/μL)	0.620 (0.542-0.698)	11.8 (7.4-18.3)	97.5 (91.3-99.6)
Hemoglobin (g/dL)	0.633 (0.558-0.708)	16.2 (10.9-23.3)	98.8 (93.3-99.9)
Platelet (10^4/μL)	0.594 (0.512-0.675)	8.1 (4.6-13.9)	95.0 (87.8-98.0)
CRP (mg/dL)	0.776 (0.713-0.839)	14.0 (9.1-20.8)	98.8 (93.3-99.9)
ESR (mm/h)	0.679 (0.606-0.753)	6.6 (3.5-12.1)	98.8 (93.3-99.9)
Albumin (g/dL)	0.785 (0.724-0.846)	6.6 (3.5-12.1)	98.8 (93.3-99.9)
CDAI	0.734 (0.668-0.800)	6.6 (3.5-12.1)	98.8 (93.3-99.9)

AUC: area under the curve; CI: confidence interval; CDAI: Crohn’s disease activity index; CRP: C-reactive protein; ESR: erythrocyte sedimentation rate; LRG: leucine-rich α-2 glycoprotein.

### LRG level and the proportion of patients below the cutoff value for each modified SES-CD category

**Figure 2.**
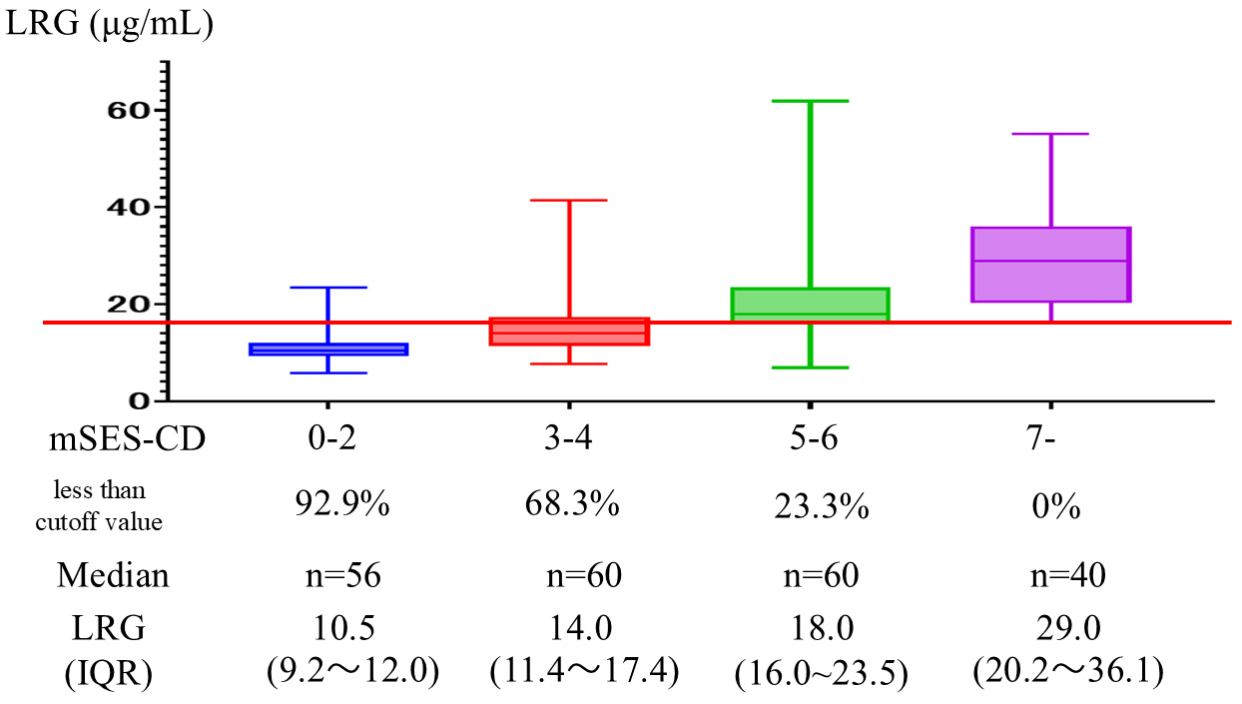
The median level of serum LRG and the percentage below the cutoff value, stratified by the mSES-CD. IQR: interquartile range; LRG: leucine-rich α-2 glycoprotein; mSES-CD: modified Simple Endoscopic Score for Crohn’s disease.

Based on the modified SES-CD, the median serum LRG level and the percentage below the cutoff value were 10.5 μg/mL (IQR, 9.2-12.0) and 92.9%, 14.0 μg/mL (IQR, 11.4-17.4) and 68.3%, 18.0 μg/mL (IQR, 16.0-23.5) and 23.3%, and 29.0 μg/mL (IQR, 20.2-36.1) and 0% for scores of 0-2 (n = 56), 3-4 (n = 60), 5-6 (n = 60), and ≥7 (n = 40), respectively (Figure 2). These results mean that all the patients in the modified SES-CD ≥7 group had LRG levels exceeding the cutoff value.

### Predictors of endoscopic activity

Independent predictors of endoscopic activity are listed in Table 3. LRG levels above the established cutoff value were strongly associated with endoscopic activity (odds ratio [OR] 36.4 [95% CI, 10.08-131.47]; p < 0.001). A higher neutrophil count was also a predictor of endoscopic activity (OR 2.91 [95% CI, 1.55-5.46]; p = 0.001). Conversely, an increased leukocyte count was negatively associated with endoscopic activity (OR 0.38 [95% CI, 0.21-0.67]; p = 0.001).

**Table 3.** Independent Predictors of Endoscopic Activity.

Variable	OR	(95% CI)	p-Value
Age (+1 year)	1.00	(0.97-1.03)	0.834
Female (vs male)	1.27	(0.41-3.92)	0.684
Body Mass Index (+1 kg/m^2^)	1.07	(0.94-1.22)	0.304
LRG ≥ cutoff value (vs < cutoff value)	36.40	(10.08-131.47)	<0.001
Leukocyte (+1,000 /μL)	0.38	(0.21-0.67)	0.001
Neutrophil (+1,000 /μL)	2.91	(1.55-5.46)	0.001
Hemoglobin (+1 g/dL)	1.05	(0.80-1.38)	0.718
Platelet (+10^4/μL)	1.00	(0.94-1.06)	0.992
CRP (+1 mg/dL)	1.35	(0.37-4.94)	0.651
ESR (+1 mm/h)	0.99	(0.95-1.04)	0.813
Albumin (+1 g/dL)	0.94	(0.68-1.30)	0.699
CDAI (+10)	1.12	(1.02-1.23)	0.016

CDAI: Crohn’s disease activity index; CI: confidence interval; CRP: C-reactive protein; ESR: erythrocyte sedimentation rate; LRG: leucine-rich α-2 glycoprotein; OR: odds ratio. ※binary logistic regression

The CDAI demonstrated a modest but significant association with endoscopic activity (OR 1.12 per 10-point increase; [95% CI, 1.02-1.23]; p = 0.016).

Other variables, including age, sex, body mass index, hemoglobin, platelets, CRP, ESR, and albumin levels, did not exhibit statistically significant associations with endoscopic activity. Thirty-six patients (16.7%) had active perianal disease. Subgroup analysis revealed no significant effect on LRG levels (p = 0.21).

### Clinical outcomes of hospitalization- and surgery-free survival

**Figure 3.**
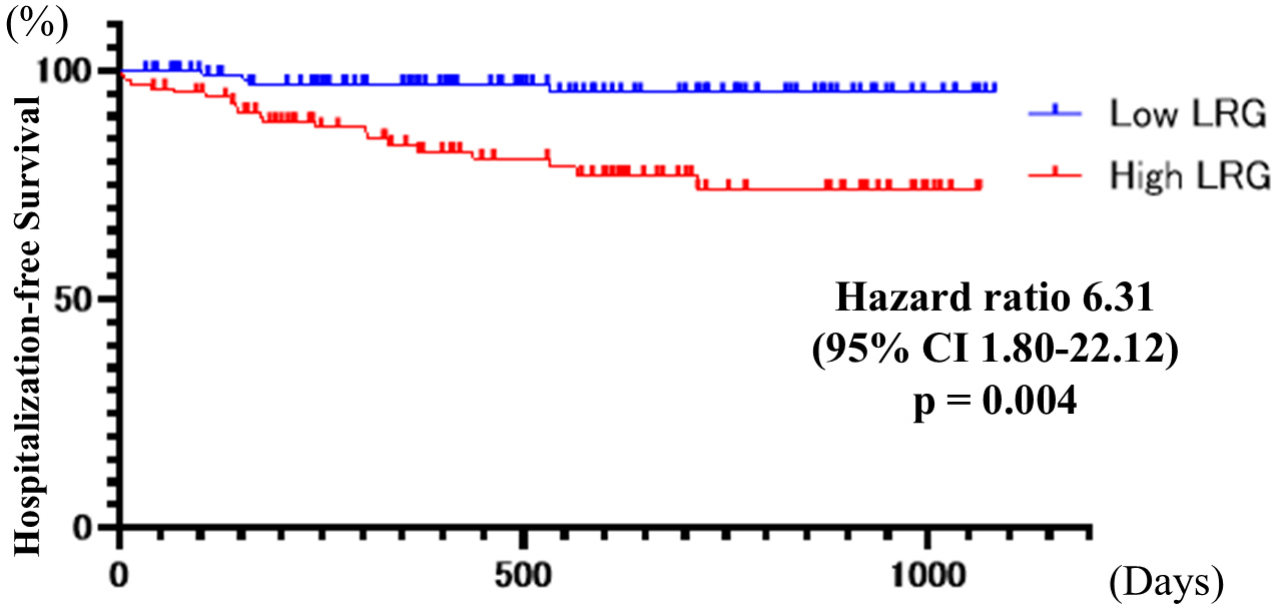
Kaplan-Meier analysis of hospitalization-free survival between low and high LRG groups. CI: confidence interval; LRG: leucine-rich α-2 glycoprotein.

**Figure 4.**
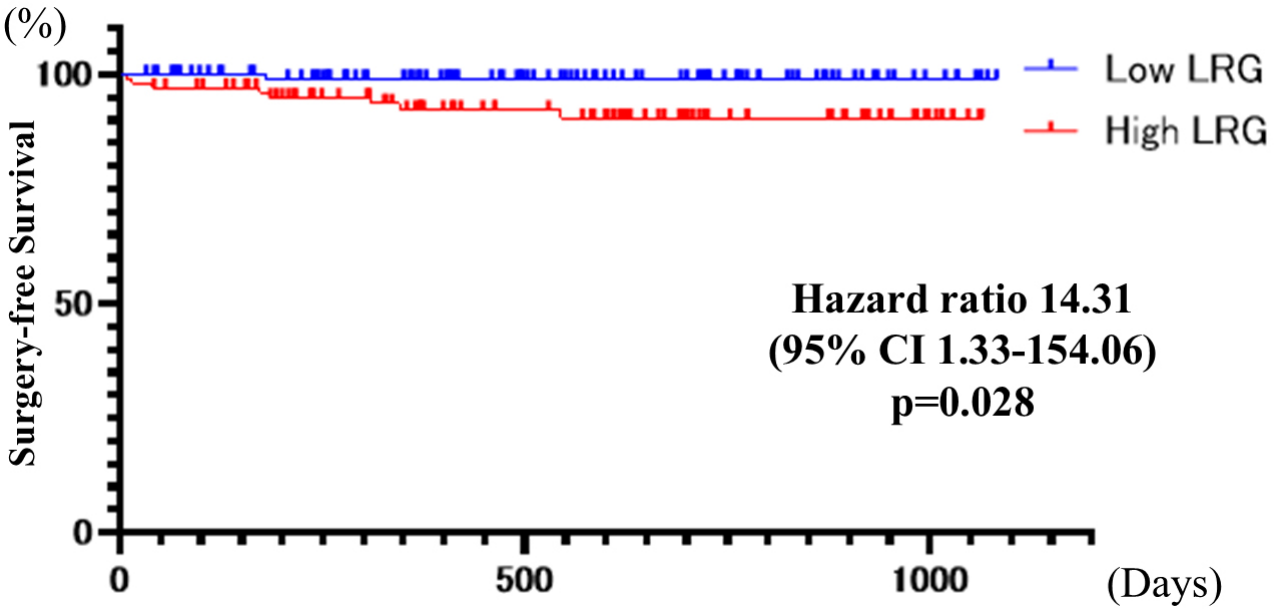
Kaplan-Meier analysis of surgery-free survival between low and high LRG groups. CI: confidence interval; LRG: leucine-rich α-2 glycoprotein.

Kaplan-Meier analysis revealed a significant association between elevated LRG levels and worse clinical outcomes. Patients with high LRG levels exhibited markedly reduced hospitalization-free survival compared to those with low LRG levels (HR 6.31 [95% CI, 1.80-22.12]; p = 0.004) (Figure 3). Similarly, surgery-free survival was significantly lower in the high LRG group (HR 14.31 [95% CI, 1.33-154.06]; p = 0.028) (Figure 4).

## Discussion

A systematic review and meta-analysis demonstrated the accuracy of LRG in assessing CD activity; however, its relationship with other biomarkers and prognosis has not yet been investigated ^(24)^. To the best of our knowledge, this is the first large-scale (>200 cases) LRG study to directly compare the utility of other biomarkers―not only CRP, but also leukocytes, neutrophils, hemoglobin, platelets, ESR, and albumin―in patients with CD and small intestinal lesions. The findings of this study underscore the potential utility of LRG as a biomarker for assessing small bowel disease activity in patients with CD. Compared with other commonly used biomarkers, LRG demonstrated superior diagnostic accuracy for predicting endoscopic activity, as evidenced by its high AUC. These results are consistent with those of previous studies highlighting the role of LRG in reflecting systemic inflammation and disease activity in CD; however, they extend this knowledge by focusing specifically on small bowel involvement. In contrast to previous reports, such as Ohno et al. ^(25)^, our study included a larger cohort and comprehensively compared LRG with a wider panel of conventional biomarkers. Furthermore, we evaluated prognostic implications using hospitalization-free and surgery-free survival, providing additional clinical value. Okita et al. ^(24)^ reported that LRG was superior to CRP in predicting mucosal healing in patients with the small intestine type compared with those with the colonic type. These results suggest that LRG may be a more useful biomarker of small intestinal lesions than of large intestinal lesions. Our study revealed that LRG was the most useful blood biomarker for small bowel disease activity in CD, even when examining a larger number of cases and a larger number of biomarkers. Neutrophils (OR 2.91, p = 0.001) and leukocytes (OR 0.38, p = 0.001) were also significantly associated with endoscopic activity.

The cutoff value for LRG established in this study (16.3 μg/mL) demonstrated both high sensitivity and specificity, providing a reliable threshold for identifying endoscopic activity. In addition, stratification of patients using the modified SES-CD revealed a clear correlation between elevated LRG levels and increased disease severity. Notably, all patients in the highest disease activity group (modified SES-CD, ≥7) exhibited LRG levels above the cutoff value. These findings suggest that LRG can serve as a robust, noninvasive marker to complement existing imaging modalities, particularly in situations where more invasive diagnostic techniques, such as BAE, are impractical or contraindicated. However, the median LRG level in our study population was 15.9 μg/mL, slightly below the established cutoff of 16.3 μg/mL, suggesting that the cohort overall may have had relatively low disease activity. This distribution bias should be considered when interpreting the generalizability of our results, and validation in populations with higher disease activity is warranted.

Several Japanese studies have reported different LRG cutoffs depending on modality and endpoint. For BAE, Kawamoto et al. reported 13.4 μg/mL for detecting ulcers ≥0.5 cm, outperforming CRP and CDAI ^(17)^. Kawamura et al. focused on endoscopic remission and identified a lower cutoff of 8.9 μg/mL with an AUC of 0.904 ^(18)^. Asonuma et al. ^(19)^ proposed dual operational thresholds (rule-in: 16 μg/mL with positive predictive value of 1.00; rule-out: 9 μg/mL with a sensitivity 0.89), highlighting use-case-dependent trade-offs. A recent multicenter BAE study targeting modified SES-CD (mSES-CD)-based endpoints yielded cutoffs of 10.5 μg/mL for endoscopic remission (mSES-CD ≤ 3) and 10.1 μg/mL for complete ulcer healing (mSES-CD ≤ 1) ^(27)^. In addition, Ohno et al. ^(25)^ corroborated the association between LRG and ileal endoscopic activity assessed with mSES-CD in BAE cohorts. Collectively, these discrepancies likely reflect differences in patient background and disease severity, endpoint definitions (ulcer detection vs remission), scoring systems (mSES-CD vs capsule-based indices), and analytical choices. Thus, our cutoff of 16.3 μg/mL should be interpreted in this methodological context and prospectively standardized across modalities.

In our cohort, 42 patients exhibited anastomotic ulcers, all of which occurred more than one year after surgery. The delayed onset suggests that these ulcers were unlikely to represent postoperative artifacts but rather reflected ongoing disease activity. These findings support considering anastomotic ulcers as clinically relevant lesions when evaluating small bowel CD activity.

Multivariate analysis identified elevated LRG level as the strongest independent predictor of endoscopic disease activity. A major strength of our study is the use of multivariate analysis in a large cohort with a wide range of biomarkers, which demonstrated that LRG is an independent factor for endoscopic activity compared with other markers.

A higher neutrophil count was independently associated with endoscopic activity. Although this finding is consistent with the role of neutrophils as key mediators of mucosal inflammation in CD, histological assessments were not performed in our study. Therefore, systemic neutrophilia does not necessarily indicate local mucosal accumulation, and further studies incorporating histopathological evaluation are warranted to clarify this relationship.

In contrast, total leukocyte counts showed a paradoxical inverse association with endoscopic activity. This discrepancy may reflect the composite nature of leukocytes, which include subpopulations less directly related to mucosal inflammation, as well as the influence of systemic factors or treatment effects. Accordingly, total leukocyte levels may have limited value as a disease-specific biomarker, whereas neutrophil counts may provide a more direct reflection of localized intestinal inflammation.

Although the CDAI showed a modest but significant association with endoscopic activity, its utility is limited by reliance on subjective symptom-based parameters. This underscores the importance of objective biomarkers such as LRG to complement clinical indices in disease assessment. Active perianal disease was observed in 16.7% of patients, but subgroup analysis demonstrated no significant effect on serum LRG levels. This suggests that LRG primarily reflects small bowel mucosal inflammation rather than perianal disease activity.

Decreased hemoglobin level, along with increased platelet count, CRP level, and ESR, are established inflammatory markers of IBD and were included in this study. However, their ability to detect endoscopic activity was poor compared with that of other markers. A study by Kawamoto ^(20)^ also included hemoglobin and platelet counts, and these biomarkers exhibited little correlation with endoscopic activity, findings that suggest these biomarkers are likely to be poorly associated with small bowel endoscopic activity.

Elevated LRG levels were significantly associated with worse clinical outcomes, including reduced hospitalization-free and surgery-free survival rates. Patients with LRG levels above the cutoff value exhibited a six-fold higher risk for hospitalization and a 14-fold higher risk for requiring surgical intervention. These findings underscore the prognostic value of LRG in identifying patients at a higher risk for adverse clinical outcomes, thereby facilitating more tailored therapeutic strategies. Mucosal healing is associated with a higher rate of steroid-free remission and reduced risks for hospitalization and surgery ^(27)^. Recent studies have emphasized that deep remission, including both mucosal healing and clinical remission, may be more significant for long-term disease management ^(28)^. Biomarkers have long been used to predict mucosal healing, and Colombel ^(10)^ reported that treatment escalation guided by biomarker levels can enhance long-term outcomes in patients with CD. For cases in which CD symptoms may not always be evident, a potential future treatment strategy could involve intensifying therapy based on LRG results. The present study had several limitations. First, it was a single-center study. Second, no histological studies were performed. Third, non-blood biomarkers, such as FC, were not evaluated at our institution because same-day results were not available. Fourth, areas not intubated using endoscopy, such as the proximal small intestine, stomach, and duodenum, were excluded. Importantly, BAE does not allow complete visualization of the entire small bowel, and undetected proximal lesions may have influenced the observed associations between LRG levels and endoscopic findings. However, because BAE enables assessment of most active areas, we believe this was unlikely to have significantly impacted the results. Fifth, the potential confounding effects of ongoing treatments, including biologics and other immunosuppressive therapies, were not accounted for in the statistical analysis. As these therapies can significantly influence LRG levels, their omission may have biased the results, and this should be considered a significant limitation. Finally, this retrospective study relied on existing medical records, which may have contained incomplete or inaccurate information, and did not permit control of confounding variables that were not documented in the data.

Incorporating LRG into routine clinical practice may improve CD management by enabling earlier identification of active disease and guiding more personalized treatment strategies. Nevertheless, given the single-center retrospective design of this study, prospective multicenter validation and comparative studies incorporating additional modalities such as MRE and capsule endoscopy are warranted to confirm and extend our findings. Although FC is globally used as a biomarker in CD, our findings suggest that LRG may provide a more practical alternative in clinical settings, as it can be measured as a serum marker at the point of care.

### Conclusions

LRG is a reliable and non-invasive biomarker for assessing small bowel disease activity and predicting adverse outcomes in CD. Incorporating LRG into clinical practice may facilitate earlier detection of active disease and support personalized treatment strategies. Prospective multicenter studies are warranted to validate these findings.

## Article Information

### Acknowledgments

The authors thank all the endoscopy medical staff at Kanazawa University Hospital, and Editage (www.editage.com) for English language editing.

### Author Contributions

Tomoyuki Hayashi conceived and designed the study, collected and analyzed the data, and drafted the manuscript. Kazuya Kitamura, Masaaki Usami, Noriaki Orita, Hidetoshi Nakagawa, Masaki Miyazawa, Hajime Takatori, Masaki Nishitani, Akihiro Dejima, Tetsuro Shimakami, Kosuke Satomura, Makiko Kimura, Hirofumi Okafuji, Hiroto Saito, Daisuke Yamamoto, and Noriyuki Inaki contributed to data collection and clinical evaluation. Tomoyuki Hayashi, and Tadashi Toyama contributed to statistical analysis. Taro Yamashita supervised the project and critically reviewed the manuscript. All authors read and approved the final manuscript.

### Conflicts of Interest

None

### Approval by Institutional Review Board (IRB)

This study was approved by the Institutional Review Board of Kanazawa University Hospital (2015-108).
